# Testing Simulation Theory with Cross-Modal Multivariate Classification of fMRI Data

**DOI:** 10.1371/journal.pone.0003690

**Published:** 2008-11-10

**Authors:** Joset A. Etzel, Valeria Gazzola, Christian Keysers

**Affiliations:** BCN Neuroimaging Center, University of Groningen, Department of Neuroscience, University Medical Center Groningen, Groningen, The Netherlands; James Cook University, Australia

## Abstract

The discovery of mirror neurons has suggested a potential neural basis for simulation and common coding theories of action perception, theories which propose that we understand other people's actions because perceiving their actions activates some of our neurons in much the same way as when we perform the actions. We propose testing this model directly in humans with functional magnetic resonance imaging (fMRI) by means of cross-modal classification. Cross-modal classification evaluates whether a classifier that has learned to separate stimuli in the sensory domain can also separate the stimuli in the motor domain. Successful classification provides support for simulation theories because it means that the fMRI signal, and presumably brain activity, is similar when perceiving and performing actions. In this paper we demonstrate the feasibility of the technique by showing that classifiers which have learned to discriminate whether a participant *heard* a hand or a mouth action, based on the activity patterns in the premotor cortex, can also determine, without additional training, whether the participant *executed* a hand or mouth action. This provides direct evidence that, while perceiving others' actions, (1) the pattern of activity in premotor voxels with sensory properties is a significant source of information regarding the nature of these actions, and (2) that this information shares a common code with motor execution.

## Introduction

The process of understanding the actions of another person, even an everyday action such as combing hair, can be performed at several levels, and potentially involves multiple interrelated systems [1]. We can understand *how* the person is performing the action (such as holding the comb in the right hand), but also *what* the person is doing (moving a comb through their hair), and *why* they are performing the action (they are getting ready for work) [1]–[3]. Simulation [4]–[8] and common coding [9], [10] models of action perception propose that we understand the *how* and *what* of other people's actions because viewing or hearing their actions activates certain brain circuits in much the same way as if we were executing the actions. While simulation and common coding models differ in particulars, especially relating to sensory perception, we will consider them as synonymous here, as they agree in the realm of action processing. Finally, the *why* of an action may be understood by theory of mind processes in an “inferential reasoning network” of cortical midline structures and the temporo-parietal junction, which may build on, interact with, or even substitute for the output of the simulation circuits [11], [12].

The discovery of mirror neurons [13], [14] has suggested a potential neural basis for the simulation and common coding models of action perception. For example, hearing someone gurgle will evoke an inner “sense” of gurgling because the brain activates some of the same mirror neurons that are active when we gurgle ourselves [14]–[16]. Given that both perceiving and executing an action is not linked to the activity of a single neuron but of a widespread population of neurons, simulation and common coding theories can be interpreted as stating that the pattern of activity while performing an action should resemble the pattern while observing or listening to a similar action. This resemblance allows the brain to interpret an activity pattern similarly whether executing or perceiving. If perception is restated as classification (was that the sound of action A or action B?) simulation theory makes a testable prediction: If the pattern of brain activity in a relevant brain region (i.e. an area with mirror neurons) is similar during action execution and perception (e.g. listening), a decision rule which determines whether action A or B was *heard* on a particular *listening* trial should also be able to determine whether action A or B was *performed* on a particular *execution* trial.

Data from single cell recordings in the monkey have been used to show that mirror neurons in the premotor cortex can indeed be used to distinguish which of two actions was executed, observed, or heard [16]. It is possible using fMRI data to compare the pattern of activity in all voxels in the premotor cortex during the perception and execution of various actions, and therefore test the predictions of the simulation theory at the population level. However, relatively few studies so far have actually measured brain activity during both the execution and the perception of multiple actions, and those that have [15], [17] have not applied methods to explicitly test whether the pattern of brain activity discriminates between actions independently of modality.

Here, we therefore used multivariate classification methods to directly test this formulation of simulation theory by determining whether a pattern classifier (a) trained to discriminate two types of actions (hand and mouth actions) using the pattern of brain activity while subjects *listened* to the sounds of these actions, could (b) classify which of these two types of actions was *executed* by the participants using the pattern of brain activity in action execution trials, patterns not presented to the classifier during training. As a proof-of-concept test to see whether cross-modal analysis is possible, we performed the analysis on a suitable existing data set [15], data previously analyzed with univariate analyses. Of the evaluated brain regions, cross-modal classification was possible only using voxels from the premotor cortex, a region thought to contain mirror neurons in humans, suggesting that this method may be valuable for testing simulation theory with fMRI data.

## Materials and Methods

### Data and Experiment

This analysis uses a portion of the data collected in an experiment investigating the human mirror neuron system in the auditory domain using univariate analyses [15]. This data set is suitable for testing the cross-modal classification strategy because two types of actions (hand and mouth) were presented in two different modalities (auditory and execution) to the same subjects. To summarize, the experiment included sixteen healthy volunteers (14 right- and two left-handed; nine female and seven male; mean age = 31 years, range = 25–45 years) with normal or corrected-to-normal vision and normal hearing. The subjects participated in several tasks, three of which are relevant here: auditory, mouth movement, and bimanual hand movement.

In the auditory task the subjects listened to the sounds of hand actions (e.g. ripping paper) and mouth actions (e.g. crunching food) as well as control sounds (e.g. water dripping). The sound recordings were four seconds long and were presented in the silent 4.1 second interval between the acquisition of volumes (acquisition time 1.5 seconds), using a T2* weighted acquisition at 3T (TE = 30 ms, TR = 5.6 s, TA = 1.5 s, 25 axial slices, 4.5 mm thick, 3.5×3.5 mm in plane resolution). Each block consisted of three stimuli of the same category, with three blocks of each type per acquisition run. Four runs were collected for each subject. For each category of sounds a total of 12 blocks (3 blocks per run for 4 runs) were therefore collected. While listening to the sounds the subjects performed an odd-ball detection task; the odd-ball was the insertion of a different category sound into a block (e.g. two mouth sound followed by a hand sound). Volumes collected during odd-ball trials were not analyzed, resulting in at most nine usable blocks for each stimulus category per subject. Only the mouth and hand sound blocks will be considered here, to match the available execution tasks.

The mouth and hand action execution tasks were performed after the auditory task and without prior warning, to avoid subject focus on performing movements while listening to the sounds. The tasks are fully described in Gazzola et. al. [15], as “MouthExe” and “HandExe.” As an overview, the mouth execution task consisted of manipulating a small plastic object (a garden-type plastic dwarf 2 cm tall) with the lips while keeping the jaw closed, while the hand execution task was to rip a piece of paper or break a peanut with both hands. 16 repetitions of each action were collected (eight each of peanut breaking and paper tearing), in different runs for each effector. The mouth execution task lasted 4 seconds, while the hand actions lasted about 5 seconds, with the duration of movement recorded and used as block length. In both cases the individual actions were separated by rest periods of 10±2 seconds, and cued visually in a single event design.

The data were temporally compressed by generating one summary volume per block. Temporal compression reduces the risk of confounding the classification with excessive block and time-dependent factors (i.e. due to the hemodynamic response lag; see also [18]). Summary volumes for each block were calculated using SPM2 (Wellcome Department of Imaging Neuroscience, London, UK; http://www.fil.ion.ucl.ac.uk/spm) by fitting a GLM with separate parameter estimates for each block. These parameter estimates were used as the measure of brain activity for each block, creating “parameter estimate images” [19], [20]. Prior to parameter estimation the volumes were high-pass filtered, realigned, and normalized (but not smoothed) as described in [15] using SPM2, except that 4 mm voxels were used; this size was chosen to have fewer voxels per ROI while maintaining the greatest possible spatial resolution given the scanning parameters. For each subject this results in 9 parameter estimate images for mouth sounds, 9 for hand sounds, 16 for mouth execution, and 16 for hand execution.

### Regions-of-Interest (ROIs)

This analysis was carried out with a region of interest (ROI)-based methodology, using all of the voxels in each ROI. The ROIs were chosen based on our simulation theory-derived hypotheses prior to analysis; brain activity measured in the subjects did not influence the choice of ROIs. Five ROIs were selected: the premotor cortex (preM) because it is seen as central to the mirror neuron system in monkeys [5], [7], [13], [14], [16] and humans [6], [8], [21]–[26]; primary (S1) and secondary somatosensory cortices (S2) [1], [6], [27]–[29], areas hypothesized to participate in somatosensory aspects of simulation; and finally the primary auditory cortex (aud) and primary motor cortex (M1), due to the nature of the tasks (auditory and motor). Each ROI was chosen individually on the left and right side. The probabilistic cytoarchitectonic maps from the SPM Anatomy Toolbox [30] were used to create the ROIs. Although the posterior parietal cortex has been implicated in the mirror neuron system, this region is not included as a ROI because of the lack of cytoarchitectonically-defined maps of the superior parietal lobule. As an exploratory analysis we analyzed a portion of the parietal lobe identified functionally, please see Text S1 for details. A further area (*other*) was included as a negative control: a comparison set of voxels which should not contain information suitable for classification of action sounds or action execution. The *other* area was made up of the early visual cortex and the amygdala, and was of comparable size to the largest ROIs (Table 1).

**Table 1:** Naming scheme and number of 4×4×4 mm voxels in each ROI and brain area.

ROI or Area	Anatomy Toolbox areas	side	abbreviation	number of voxels	
				total	without somatotopic
premotor cortex	BA 44, BA 6	left	preM L	396	386
		right	preM R	385	376
auditory cortex	TE 1.0, TE 1.1, TE 1.2	left	aud L	55	
		right	aud R	61	
secondary somatosensory cortex	OP1, OP2, OP3, OP4	left	S2 L	159	
		right	S2 R	162	
primary motor cortex	BA 4a, BA 4p	left	M1 L	183	
		right	M1 R	147	
primary somatosensory cortex	BA 1, BA 2, BA 3a, BA 3b	left	S1 L	262	
		right	S1 R	351	
*other*	CM/LB/SF; BA 17, BA 18, hOC5	left	*other* L	391	
		right	*other* R	348	

The given voxel counts are the number of voxels used in the analyses (the number that remain after removing all voxels with zero variance across volumes in any subject), both in each ROI and after removing somatotopic voxels; see text for details. The “Anatomy Toolbox areas” column lists the regions selected to make up each ROI or area using the names in the probabilistic cytoarchitectonic maps from the SPM Anatomy Toolbox [30]. See Figure S1 and Figure S2 for an illustration of these ROIs.

The voxels in each ROI were further processed to exclude voxels which had zero variance across parameter estimate images in any subject because voxels with zero variance cannot contribute classification information. Including only voxels with non-zero variance in all subjects ensures that each ROI is the same size in all subjects, facilitating the comparison of results across subjects, but potentially eliminating voxels which contain classification information in a subset of subjects. The ImCalc function in SPM2 was used to transform the masks to the same shape and voxel size as the parameter estimate images. The number of voxels and specific SPM Anatomy Toolbox areas composing each ROI, as well as *other*, are listed in Table 1. The ROIs are shown in Figure S1.

### Classification Procedure

Support vector machines (svms) [31], [32] were used as the classifier for this analysis. All analyses were performed in R [33], using the e1071 package's svm command with a linear kernel, cost = 1, and default scaling (to zero mean and unit variance). These choices are similar to those previously used with fMRI data [34]–[39]. The classification was done within-subjects, with the results averaged across subjects. For cross-modal classification a classifier was trained to distinguish mouth and hand sounds for each subject and ROI separately, using the nine sound parameter estimate images for each condition as the training set. The classifiers were then presented with execution data to classify as the test set; no execution task data were used during training. The proportion of correctly classified execution parameter estimate images was used as the classification accuracy for the subject and ROI. Stated another way, the classifiers determined whether an execution trial was more similar to the hand or mouth sound activation pattern.

Given that only ∼10% of neurons in the premotor cortex respond to the sound of actions [14], [16] while virtually all respond during the execution of actions, training a classifier on auditory data and testing it on execution data is preferable to the reverse procedure because it ensures that the classifiers focus on the subset of voxels that do contain sensory information (and are therefore likely to be mirror). The results, therefore, do not show that the activity pattern of a cortex overall is similar during action execution and perception, but that those voxels with sensory properties show an activity pattern that is similar during perception and action.

The significance of the classification accuracy for each ROI was determined by a permutation test which determines how likely it is to get an accuracy as high as the one observed (similar to the procedure described in [40]). This is done by testing the null hypothesis that there is no relationship between the test data class labels (mouth or hand) and the voxel activity pattern. A lack of relationship is ensured by randomly permuting the test data labels. There are too many possible permutations to do a complete permutation test (_32_C_16_≅10^8^), so a random permutation test was performed by calculating the accuracy of 1000 random data relabelings (i.e. randomly reordering the “mouth” and “hand” execution labels, using the same ordering for each subject). The classification accuracy of each relabeled data set is determined in the same manner as for the actual data set, and the average across-subjects accuracy computed. The *p*-value is then determined by counting the proportion of relabeled data sets classified more accurately than the true data set. As 1000 relabelings were computed the maximum significance level possible is 1/1001 = 0.001 [41], [42]. Significance was evaluated by t-tests as well, by evaluating the likelihood that the true overall mean accuracy of the 16 subjects is greater than 0.5 (chance level). In our opinion the permutation test is more appropriate for this data since it does not require distributional assumptions and directly tests the hypothesis of interest, so permutation test *p*-values will be used in the text, although t-test *p*-values are also reported for interested readers.

In addition to the cross-modal classification, uni-modal classification was performed by classifying the data from the sound trials alone. Uni-modal classification indicates how accurately each ROI was able to distinguish the sound data. In brief, this was carried out by making training sets of all but one mouth and all but one hand action sound block per subject, with the remaining two examples used as the test set. Every example was used once in a test set (stratified nine-fold cross-validation), pairing test samples collected closest together in time. Performance was quantified as the test set accuracy, averaged over the nine test sets for each subject. Significance was calculated by a complete permutation test (performed pairwise in order to maintain stratification) and a one-sided t-test (true mean greater than 0.5), as for the cross-modal analysis.

## Results

Of the ten ROIs, significant (*p*<0.005) cross-modal classification was possible only using the left and right premotor cortex (Table 2). Examining the classification performance of the ROIs using the auditory data alone (Table 3) shows that the superior classification accuracy of the premotor cortex was specific for cross-modal classification: several ROIs, auditory in particular, classified sounds more accurately than premotor, but their higher accuracy did not carry across modalities. The highest uni-modal classification accuracy was obtained in aud L; aud R, S2 L, S2R, preM L, preM R, and S1 L, could also classify significantly above chance (Table 3). The patterns in M1 L, M1 R, and S1 R could not be classified.

**Table 2:** Mean cross-modal classification accuracy and *p*-values of each ROI as determined by permutation and t-testing, both of the entire ROI and after removing the voxels identified as somatotopic; see text for details.

ROI	all voxels				without somatotopic voxels			
	mean	s.e.m.	perm *p*-value	t-test *p*-value	mean	s.e.m.	perm *p*-value	t-test *p*-value
preM L	0.5449	0.0232	0.005*	0.0358	0.543	0.023	0.0040*	0.0406
preM R	0.5664	0.0197	0.001*	0.0021*	0.5586	0.0215	0.0030*	0.0078
M1 L	0.4727	0.0299	0.9441	0.8125				
M1 R	0.4863	0.0331	0.7343	0.6571				
S1 L	0.5352	0.0224	0.043	0.069				
S1 R	0.5059	0.0225	0.3866	0.3991				
S2 L	0.5176	0.0213	0.1788	0.2115				
S2 R	0.5156	0.0278	0.2517	0.2912				
aud L	0.5508	0.0238	0.014	0.0251				
aud R	0.5312	0.0278	0.0679	0.1394				

* *p*<0.0050 (Bonferroni correction of 0.05 for 10 ROIs).

**Table 3:** Mean uni-modal (train and test on listening data) classification accuracy.

ROI	mean	s.e.m.	permutation *p*-value	t-test *p*-value
preM L	0.5729	0.0478	0.002*	0.0739
preM R	0.5729	0.0382	0.002*	0.0378
M1 L	0.5521	0.0364	0.0176	0.0864
M1 R	0.5069	0.0408	0.1037	0.4336
S1 L	0.6181	0.0464	0.002*	0.0113
S1 R	0.5174	0.0453	0.0607	0.3534
S2 L	0.5868	0.0336	0.002*	0.0104
S2 R	0.625	0.0435	0.002*	0.0058
aud L	0.6389	0.0296	0.002*	0.0001*
aud R	0.5938	0.0294	0.002*	0.0031*

* *p*<0.0050 (Bonferroni correction of 0.05 for 10 ROIs).

The *other* areas were included to serve as a comparison set of voxels which should not contain information suitable for classification of action sounds or action execution. *Other* serves as a sort of negative control, to check that cross-modal classification accuracy is not simply something that is possible in any group of voxels from this data. It is possible that some stimulus-relevant activity exists in these areas, due to cross-talk with other brain regions. This does not diminish their value as a negative control if the classification accuracy of these regions is indistinguishable from chance, however, which is what resulted from both the uni-modal and cross-modal analyses (Table 4).

**Table 4:** Mean uni-modal (train and test on listening data) and cross-modal (train on listening, test on execution) classification accuracy and *p*-values of the *other* areas.

area	analysis	mean	s.e.m.	permutation *p*-value	t-test *p*-value
*other* L	uni-modal	0.5382	0.0426	0.0254	0.1923
	cross-modal	0.5332	0.0201	0.0549	0.0594
*other* R	uni-modal	0.4618	0.0242	0.3366	0.9325
	cross-modal	0.5332	0.0227	0.0559	0.0823

The previous group analysis of the data from this experiment [15] provided evidence for a somatotopic mirror neuron system, portions of which overlap the ROIs considered here. If classification accuracy remains significant when the voxels shown to have somatotopic properties in the group analysis are excluded, we have evidence that cross-modal multivariate pattern classification relies upon additional sources of information: patterns present in voxels previously not identified as containing significant information. To investigate this possibility the voxels identified as somatotopic in [15] (using mass-univariate analysis at the group level) were removed from the premotor ROIs and the cross-modal analysis was repeated. Specifically, the voxels shown in yellow and red in Figure S4 frame D of [15] were removed (see Figure S2). This is a larger group of voxels than that used in the primary results of [15] since they meet a less stringent definition of somatotopy, and so provide a more difficult classification challenge. The number of voxels remaining in the left and right premotor ROIs after removing these somatotopic voxels is listed in Table 1. Removing these voxels had little effect on the cross-modal classification accuracy (Table 2): significant (*p*<0.005) cross-modal classification was still found in the left and right premotor cortex.

## Discussion

The main result of this report is that cross-modal classification was possible: pattern classifiers could determine whether an executed action involved the hand or the mouth using the pattern of brain activity in the premotor cortex after having been trained to discriminate the activity pattern while subjects listened to the sound of hand and mouth actions. Since the classifiers were trained on the sound data this does not show that the activity pattern of the premotor cortex as a whole is similar during action execution and perception, but rather that those voxels with sensory properties show an activity pattern that is similar during perception and action. Of the ROIs tested, only the premotor cortex had significant cross-modal classification, which informs the debate of whether motor or somatosensory simulation dominates social perception [14]–[16], [43]–[45]. This shows that one of the core predictions of simulation and common coding theories is correct: a pattern classifier that has learnt to decode which action was heard based on the pattern of brain activity in the premotor cortex during action perception trials *can* successfully deduce which action was executed in action execution trials. In particular, these results show that multivariate pattern classifiers can be used to study simulation and common coding theories. Whether areas outside of the ROIs tested here are capable of classifying actions across modality, and might therefore also participate in simulation and common coding, remains a question for future research, with the parietal cortex being an obvious candidate (see Text S1).

However, we need to consider what it means to find a classification accuracy of 57% correct with *p*<0.005 (threshold of 0.05, Bonferroni-corrected for ten ROIs). Humans can discriminate hand and mouth actions much more accurately than this, typically >90% correct [15]. Does that mean that the premotor cortex cannot be the neural basis for such discrimination? In mass-univariate fMRI analysis the absolute difference between two conditions is always minimal (typically less than 1% of the BOLD signal), but if the difference is unlikely to have occurred by chance (*p*<0.05 corrected for multiple comparison) a brain region is considered to be involved in the task. The fact that the difference is small in absolute terms is unsurprising because fMRI is only a very indirect and noisy measure of neural activity. Translated to multivariate analysis, significant above-chance classification, independently of its absolute value, should therefore be considered to provide similarly meaningful evidence that the region has task-relevant information–but at the level of patterns of activity. This is why recent reports [e.g. 20], [46] consider the significance of classification accuracies more important than their absolute values: because BOLD is such an indirect measure of neural activity. The fact that the absolute value of classification accuracy is lower than that of humans simply reflects the degradation of the neural signal along the causal chain of fMRI measurements. In this specific case, the degradation is especially severe because cross-modal action classification suffers from two additional problems. First, mirror neurons preferring hand actions are sometimes recorded so closely to those preferring mouth actions that their activity could cancel each other out within the volume of our fMRI voxels [16]. Second, only about 10% of motor neurons are mirror [14], [16] and classifiers, unlike the brain, which may somehow focus on the activity in these mirror neurons, therefore have to pick the 10% mirror ‘signal’ out of the 90% non-mirror ‘noise’. In light of these considerations, above-chance classification across modality based on fMRI signals should be seen as an experimental proxy to examine whether there is evidence for patterns of activity at the neural level that would support even more reliable classification. It is hoped that even greater significance, and perhaps higher classification accuracy levels, may be possible when analyzing data from experiments specifically designed to test cross-modal classification. We are currently beginning such an experiment.

This report describes an analysis technique which we believe can provide the most direct support for simulation [4]–[8] and common coding [9], [10] theories possible with fMRI. Classical univariate analysis using the same data [15] could only show that certain voxels ‘preferred’ the same type of actions (hand or mouth) both during listening and execution. This finding leaves unanswered the essential question of whether the brain could use this information to perceive which action another individual is performing at a particular point in time in terms of the listener's own actions. One reason is that, while differences are present at the group level, activity in other voxels could obscure the information at the individual level, for instance by responding more to hand actions during listening and more to mouth actions during execution. An additional limitation for interpretation is that for an individual to perceive actions accurately requires that the activity pattern induced by action perception is reliable from trial to trial, whereas traditional group analyses focus on determining whether activity is similar from person to person (averaging across trials at the first level of analysis). Here, separate multivariate classifiers were trained for each individual, thus identifying patterns which were similar across trials for that individual. Cross-modal classification directly indicates that the *pattern* of activity in the premotor cortices, with all its individual peculiarities, is similar enough during the execution and perception of actions–at least in voxels with sensory properties since the classifiers were trained on the auditory data–to provide the listener with a way to perceive the actions of others through his/her own actions. The multivariate approach thus provides a fundamental advantage over mass-univariate approaches for this purpose, and we hope that the present paper, using pattern classification to investigate simulation theories, will prove to be a powerful new tool to investigate the idea of common coding at the level of neural populations as measured using fMRI.

## Supporting Information

Text S1 Cross-Modal Classification of a Parietal Region.
(0.12 MB DOC)

Figure S1 The ROIs in a glass brain representation, rendered on the mean anatomy of the 16 subjects with maximum transparency depth. The ROIs on each side are shown with the same color for clarity, although always analyzed separately on the left and right sides. See Table 1 for the derivation and size of each ROI.
(1.02 MB TIF)

Figure S2 Slices showing the voxels omitted from the premotor cortex, superimposed on the mean anatomy; see the Results for a description of the procedure. The slice numbers are given first as analyzed (4 mm×4 mm×4 mm voxels), followed by the Talairach coordinate slice numbers in parentheses. Starting at the upper left and moving left to right, these are slices z = 14 (54 to 58), z = 17 (67 to 69), z = 24 (94 to 98), z = 25 (99 to 101), z = 29 (115 to 117), and z = 30 (118 to 122).
(2.03 MB TIF)
