# Supplementary material for: Testing Simulation Theory with Cross-Modal Multivariate Classification of fMRI Data
Source: PLoS One. 2008 Nov 10;3(11):e3690. doi: 10.1371/journal.pone.0003690 (PMC2577733; doi:10.1371/journal.pone.0003690)
Supplement: Text S1 — Cross-Modal Classification of a Parietal Region. (0.12 MB DOC) [file pone.0003690.s001.doc]

# Text S1. Cross-Modal Classification of a Parietal Region.

The posterior parietal cortex has been implicated in the mirror neuron system [1-3], and the reader may wonder whether cross-modal multivariate classification is possible in parietal regions. However, the posterior parietal cortex was not included as a ROI in the main manuscript due to the lack of cytoarchitectonic maps of this lobe.

Nevertheless, we created a posterior parietal ROI for exploratory purposes by combining functional and cytoarchitectonic data with anatomical knowledge. The functional data came from a separate study [4] in which the same participants used here *viewed* movies of hands manipulating objects, viewed static images of hands and objects, and executed hand actions. In this study voxels were considered part of the putative MNS if they were (1) more active while viewing manipulation than static images of the hand and objects, and (2) more active during the execution of hand actions compared to a passive baseline (see [4] for further details). This step identified active voxels in the posterior parietal lobe, but also in other brain regions. The MarsBar toolbox for SPM2 [5] was used to identify clusters appearing, based on our anatomical knowledge, to fall in the parietal lobe. These voxels were masked exclusively with the S1 and S2 ROIs, so that no voxel could be assigned to both the parietal ROI and S1 or S2. These steps result in a parietal ROI with 82 voxels on the right and 159 on the left, using 4 mm x 4 mm x 4 mm voxels as in the main analysis; see Figure S3 for a depiction of the ROI.

The cross-modal classification accuracy of this parietal ROI is not directly comparable with the accuracy of the ROIs because of this different method of construction. Particularly, we restricted this parietal area to voxels which showed significant activity in [4] (using mass-univariate analyses), whereas *all* voxels were included in the other ROIs. Restricting the area to voxels in this way may improve the classification performance (e.g. [6,7]). We therefore do not include these results in the main text, to avoid misleading comparisons between the accuracy of the hybrid posterior parietal ROI and the other, purely anatomical, ROIs.

The cross-modal classification results of the parietal areas are shown in Table S1. As for the other ROIs (Table 2), significance was assessed by both a permutation and t-test. The left parietal area did not classify significantly better than chance, while the right region was significantly better, at mean accuracy of 0.57, *p* = 0.001 (permutation test). This exploratory analysis can be considered an indication that cross-modal classification may be possible in this region as well.

**Table S1. Mean cross-modal classification accuracy and p-values of each ROI as determined by permutation and t-testing for the parietal area.**

| **Area** | **Mean** | **s.e.m.** | **perm**  ***p*-value** | **t-test**  ***p*-value** |
| --- | --- | --- | --- | --- |
| parietal L | 0.5215 | 0.0166 | 0.1828 | 0.1071 |
| parietal R | 0.5664 | 0.0211 | 0.001 | 0.0034 |


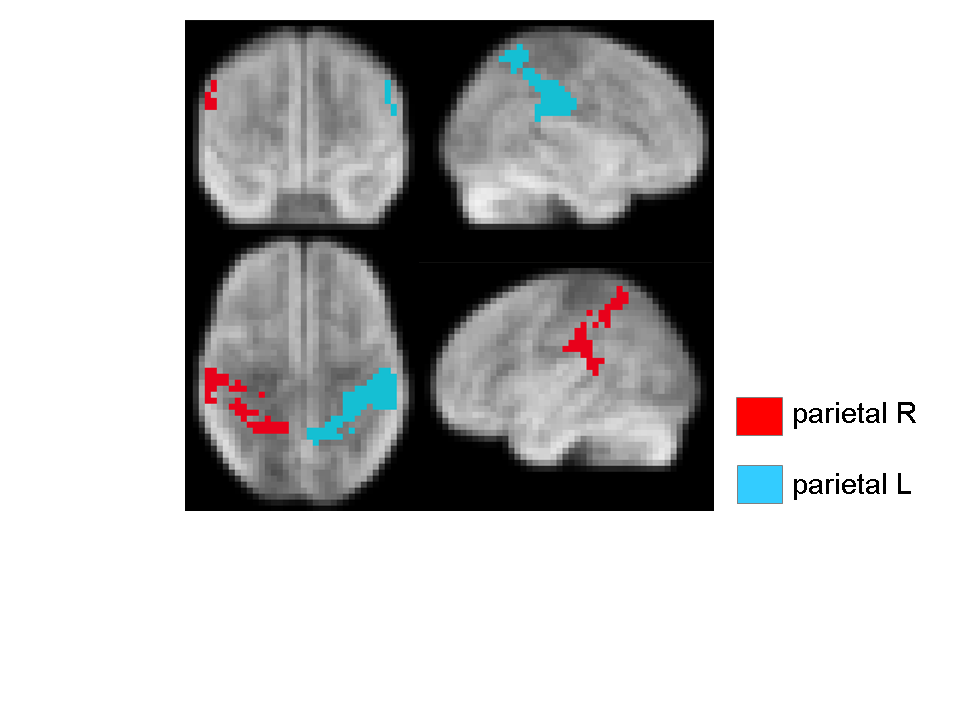


Figure S3. The left and right parietal ROIs, rendered on the mean anatomy of the 16 subjects, with transparency depth of 9 voxels (24 mm). See Text S1 for the derivation and size of this ROI.

## References

1. Rizzolatti G, Craighero L (2004) The mirror-neuron system. Annu Rev Neurosci 27: 169-192.

2. Iacoboni M, Molnar-Szakacs I, Gallese V, Buccino G, Mazziotta JC, et al. (2005) Grasping the Intentions of Others with One's Own Mirror Neuron System. PLoS Biology 3: e79.

3. Buccino G, Binkofski F, Fink GR, Fadiga L, Fogassi L, et al. (2001) Action observation activates premotor and parietal areas in a somatotopic manner: an fMRI study. European Journal of Neuroscience 13: 400-404.

4. Gazzola V, Rizzolatti G, Wicker B, Keysers C (2007) The anthropomorphic brain: The mirror neuron system responds to human and robotic actions. Neuroimage 35: 1674-1684.

5. Brett M, Anton JL, Valabregue R, Poline JB. Region of interest analysis using the MarsBar toolbox for SPM 99; 2002; Sendai, Japan. Available on CD-ROM in NeuroImage, Vol 16, No 2.

6. Mitchell TM, Hutchinson R, Niculescu RS, Pereira F, Wang X (2004) Learning to Decode Cognitive States from Brain Images. Machine Learning 57: 145-175.

7. Cox DD, Savoy RL (2003) Functional magnetic resonance imaging (fMRI) "brain reading": detecting and classifying distributed patterns of fMRI activity in human visual cortex. Neuroimage 19: 261-270.
