# Supplementary figures and images for: Testing Simulation Theory with Cross-Modal Multivariate Classification of fMRI Data
Source: PLoS One. 2008 Nov 10;3(11):e3690. doi: 10.1371/journal.pone.0003690 (PMC2577733; doi:10.1371/journal.pone.0003690)

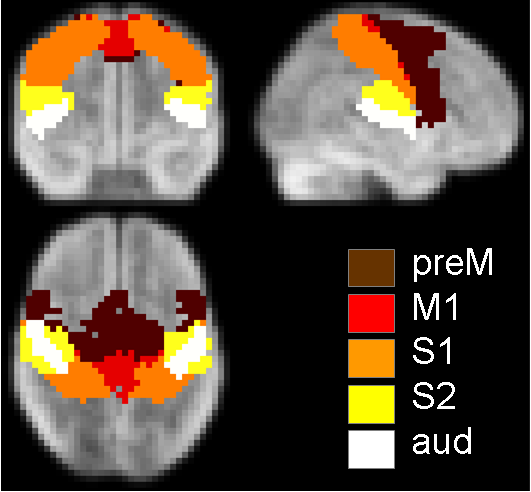

Supplement: Figure S1 — The ROIs in a glass brain representation, rendered on the mean anatomy of the 16 subjects with maximum transparency depth. The ROIs on each side are shown with the same color for clarity, although always analyzed separately on the left and right sides. See Table 1 for the derivation and size of each ROI. (1.02 MB TIF) [file pone.0003690.s002.tif]

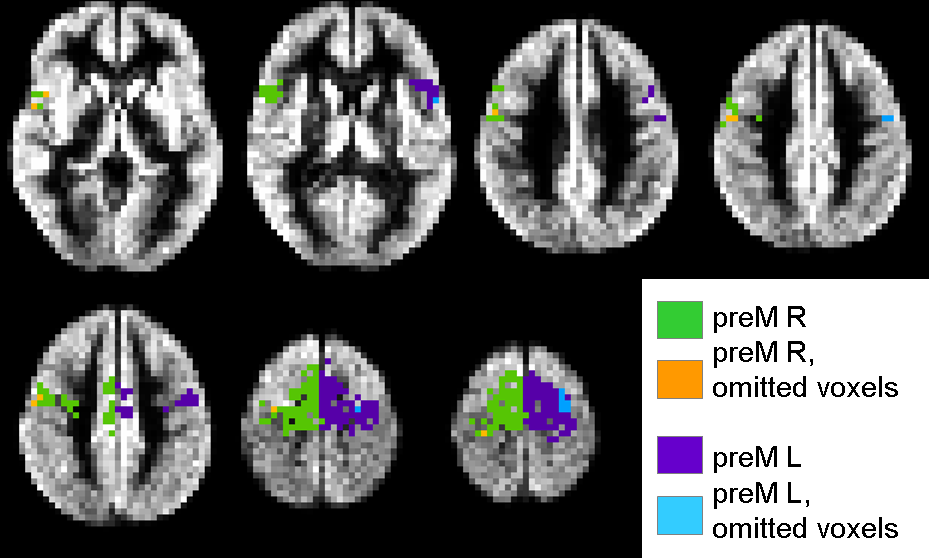

Supplement: Figure S2 — Slices showing the voxels omitted from the premotor cortex, superimposed on the mean anatomy; see the Results for a description of the procedure. The slice numbers are given first as analyzed (4 mm×4 mm×4 mm voxels), followed by the Talairach coordinate slice numbers in parentheses. Starting at the upper left and moving left to right, these are slices z = 14 (54 to 58), z = 17 (67 to 69), z = 24 (94 to 98), z = 25 (99 to 101), z = 29 (115 to 117), and z = 30 (118 to 122). (2.03 MB TIF) [file pone.0003690.s003.tif]
